# What’s in a label? The importance of clinical specificity from the perspective of patients with primary progressive apraxia of speech

**DOI:** 10.1002/alz.084546

**Published:** 2025-01-09

**Authors:** Rene L Utianski, Gabriela Meade, Joseph R Duffy, Heather M. Clark, Hugo Botha, Jennifer L. Whitwell, Keith A. Josephs

**Affiliations:** ^1^ Mayo Clinic, Rochester, MN USA

## Abstract

**Background:**

Discussion surrounding the nomenclature of the “nonfluent/agrammatic” spectrum of progressive speech‐language disorders has largely focused on the clinical‐pathological and neuroimaging correlations, with some attention paid to the prognostication afforded by differentiating clinical phenotypes. Progressive apraxia of speech (AOS), with or without agrammatic aphasia, is generally associated with an underlying tauopathy; however, patients have offered a unique perspective on the importance of distinguishing between difficulties with speech and language that extends beyond pathological specificity. This study aimed to provide insight into the experience of patients with primary progressive AOS (PPAOS), with particular attention to their diagnostic journey.

**Method:**

A qualitative research approach was used. Structured interviews were conducted with three prosodic and one phonetic predominant PPAOS patients, judged representative of our research cohort, who were previously received other diagnoses, including primary progressive aphasia (PPA). A current PPAOS diagnosis and willingness to discuss the details of their diagnostic workup and reflect upon their experience were the inclusion criteria. One patient preferred to provide written responses. Each interview was reviewed, and the collective contents were examined using thematic analysis.

**Result:**

The primary themes were identity, community, and planning. Patients expressed: 1) the importance of the alignment of AOS with their difficulties, specifically that they had no difficulty with thinking of words or with comprehension (i.e., no aphasia); 2) not sharing attributes with PPA patients and their observed clinical presentations; and 3) the desire to capitalize upon their retained skills for treatment, for example **“**I intend to retain my ‘voice’ even as I lose my speaking ability.”

**Conclusion:**

Understanding the experiences of PPAOS patients may inform future iterations of progressive speech‐language spectrum diagnostic criteria and the consistency of their use, along with intervention recommendations. The specificity of a PPAOS diagnosis is meaningful to patients as it relates to their identity, sense of community, and approach to future planning. Further work will evaluate the experience of patients with a broader spectrum of progressive speech‐language disorders along with their care partners. More work is underway to outline the predictive value of an AOS diagnosis, relative to co‐occurring aphasia severity, to inform better prognostication.

